
JOURNAL INFORMATION
==============================
NLM Title Abbreviation: Intensive Care Med Exp
Journal ID: Intensive Care Med Exp
NLM Journal ID: 101645149

Intensive Care Medicine Experimental

EISSN: 2197-425X
Publisher: Springer

ARTICLE INFORMATION
==============================
PMCID: PMC8503872
PMID: 34633571
DOI: 10.1186/s40635-021-00415-6
Article ID: 415
Article version: 2
Subjects: Meeting Abstracts

ESICM LIVES 2021: Part 2

Electronic publication date: 2021 Oct 11
Publication date: 2021 Oct
Volume: 9
Issue: Suppl 1
Issue sponsor: Publication of this supplement has not been supported by sponsorship.
Electronic Location ID: 50
Copyright: © The Author(s) 2021
Copyright year: 2021
License: Open Access This article is licensed under a Creative Commons Attribution 4.0 International License, which permits use, sharing, adaptation, distribution and reproduction in any medium or format, as long as you give appropriate credit to the original author(s) and the source, provide a link to the Creative Commons licence, and indicate if changes were made. The images or other third party material in this article are included in the article’s Creative Commons licence, unless indicated otherwise in a credit line to the material. If material is not included in the article’s Creative Commons licence and your intended use is not permitted by statutory regulation or exceeds the permitted use, you will need to obtain permission directly from the copyright holder. To view a copy of this licence, visit http://creativecommons.org/licenses/by/4.0/.
License URL: https://creativecommons.org/licenses/by/4.0/

Conference name: 34th ESICM Annual Congress
Conference Acronym: ESICM LIVES 2021
Conference location: Virtual
Conference date: 03-06 October 2021
issue-copyright-statement: © European Society of Intensive Care Medicine and Springer Nature Switzerland AG 2021

==============================
Publisher's Note

Springer Nature remains neutral with regard to jurisdictional claims in published maps and institutional affiliations.

